# Assessing the susceptibility and efficacy of traditional neurotoxic (pyrethroid) and new-generation insecticides (chlorfenapyr, clothianidin, and pyriproxyfen), on wild pyrethroid-resistant populations of *Anopheles gambiae* from southern Benin

**DOI:** 10.1186/s12936-023-04664-6

**Published:** 2023-08-26

**Authors:** David Mahouton Zoungbédji, Germain Gil Padonou, Alphonse Keller Konkon, Steve Hougbe, Hermann Sagbohan, Casimir Kpanou, Albert Sourou Salako, Razaki Ossè, Rock Aïkpon, Cyriaque Afoukou, Aboubakar Sidick, Bruno Akinro, Saïd Chitou, Virgile Gnanguénon, Patrick Condo, Ahmed Saadani Hassani, Daniel Impoinvil, Martin Akogbéto

**Affiliations:** 1 Centre de Recherche Entomologique de Cotonou (CREC), 06 BP 2604 Cotonou, Benin; 2 Faculté des Sciences et Techniques, Université d’Abomey-Calavi, Godomey, Benin; 3 Programme National de Lutte Contre Le Paludisme, Cotonou, Benin; 4 US President’s Malaria Initiative, US Agency for International Development, Cotonou, Benin; 5 US President’s Malaria Initiative, US Centers for Disease Control and Prevention, Cotonou, Benin; 6 US President’s Malaria Initiative, US Centers for Disease Control and Prevention, Atlanta, USA

**Keywords:** Chlorfenapyr, Pyriproxyfen, Clothianidin, *Anopheles gambiae*, Insecticide resistance, Long-lasting insecticidal nets, Bio-efficacy, Benin

## Abstract

**Background:**

The objective of this study was to determine the susceptibility of wild *Anopheles gambiae *sensu lato (*s.l*.) from southern Benin to the new insecticides (chlorfenapyr (CFP), pyriproxyfen (PPF), and clothianidin (CTD)) and assess the efficacy of insecticide-treated bed nets (ITNs) that contain these new products.

**Methods:**

Wild *An. gambiae* from the Benin communes of Allada, Ifangni, Akpro-Missérété, and Porto-Novo were tested for their susceptibility to CFP and PPF using the WHO bottle tests, and pyrethroids (alpha-cypermethrin, deltamethrin, and permethrin) and CTD using WHO tube tests. WHO cone tests were used to evaluate the efficacy of Interceptor^®^ (which contains alpha-cypermethrin (ACM) only), Interceptor^®^ G2, (CFP + ACM), and Royal Guard^®^ nets (PPF + ACM). The ovaries of blood-fed *An. gambiae* from Ifangni exposed to a new PPF net were dissected, and egg development status was examined using Christopher’s stages to determine the fertility status of the mosquitoes. Using a standardized protocol, the oviposition rate and oviposition inhibition rate were calculated from live blood-fed *An. gambiae* placed in oviposition chambers after exposure to PPF.

**Results:**

In all four mosquito populations, pyrethroid mortality ranged from 5 to 80%, while chlorfenapyr and clothianidin mortality ranged from 98 to 100%. At Ifangni, all mosquitoes exposed to Royal Guard® nets were infertile (100%) while the majority (74.9%) of mosquitoes exposed to Interceptor® nets had fully developed their eggs to Christopher’s stage V. The oviposition inhibition rate after exposure of the mosquitoes to the PPF was 99% for the wild population of *An. gambiae s.l.* and the susceptible laboratory strain, *An. gambiae *sensu stricto (Kisumu).

**Conclusions:**

The results of this study suggest that pyrethroid-resistant *An. gambiae* from the selected communes in southern Benin are susceptible to chlorfenapyr, clothianidin, and pyriproxyfen. In addition, based on bioassay results, new and unused Interceptor® G2 and Royal Guard® nets were effective on Ifangni’s mosquito populations. Despite the availability of new effective insecticides, continued vigilance is needed in Benin. Therefore, monitoring of resistance to these insecticides will continue to periodically update the Benin national insecticide resistance database and management plan.

## Background

Long-lasting insecticidal nets (LLINs) are widely used as a preventive measure for controlling malaria in sub-Saharan Africa (SSA) [1]. Their massive scale-up has led to a major reduction in the malaria burden in many sub-Saharan African countries [2, 3]. Thus, the distribution of insecticide-treated nets (ITNs) started in Benin, in 2000. The switch to LLINs as a core vector control intervention distributed to targeted groups occurred in 2005. Thereafter, mass distribution campaigns were performed every 3 years since 2011, with the aim of providing at least one net for every two people in a household. The World Health Organization (WHO) estimates that as of 2021, 54% of the SSA population has access to an LLIN and 47% are sleeping under an LLIN [4]. The ownership rate has increased to 65% since 2020. However, the rapid spread of pyrethroid-resistant vectors seriously threatens to reverse the gains made [5–7]. Indeed, several studies have shown that LLINs become less effective at killing mosquitoes in areas of high resistance compared to areas of susceptibility [8, 9]. It is not clear the extent to which insecticide resistance has contributed to the malaria burden, but it is worth noting that the global malaria cases continued to rise to 232 million in 2019, 245 million in 2020, and 247 million in 2021[4]. Therefore, the WHO supports the development of alternative tools combining multiple insecticides to improve vector control and insecticide resistance management [10].

To maintain the effectiveness of vector control tools, products with new insecticides including chlorfenapyr (CFP), clothianidin (CTD), and pyriproxyfen (PPF) are outlined on the WHO list of Prequalified Vector Control Products. Clothianidin, a neonicotinoid insecticide, is a relatively new insecticide being used in indoor residual spraying (IRS) with new products including Fludora^®^ Fusion, SumiShield^®^ Klypson™, and 2GARD™. Conversely, new nets with dual active ingredients have emerged such as Interceptor^®^ G2 nets with alpha-cypermethrin and chlorfenapyr, a pyrrole insecticide, and Royal Guard^®^ nets with alpha-cypermethrin and pyriproxyfen, an insect growth regulator.

Clothianidin acts as an agonist of nicotinic acetylcholine receptors causing paralysis and death in insects [11, 12]. Chlorfenapyr disrupts the energy pathways of insects in their mitochondria by preventing the formation of adenosine triphosphate (ATP), resulting in cell death and insect mortality [13]. Pyriproxyfen causes insect-specific physiology and morphogenesis [14] changes in adult mosquitoes by disrupting ovarian development, egg laying, and hatching [15, 16]. Clothianidin is very effective against mosquito vectors in laboratory [17, 18], semi-field [19], and field trials [20, 21]. Chlorfenapyr and pyriproxyfen products have demonstrated superior efficacy compared to the standard pyrethroid LLIN in small-scale entomological trials and were found to be effective in Phase I and Phase II trials in West Africa [22–26]. There are also ongoing studies in Benin [27, 28] and Tanzania [29] on the efficacy of two dual-active ingredient long-lasting insecticidal nets (Royal Guard® and Interceptor® G2) for control of malaria transmitted by pyrethroid-resistant vectors.

As part of the routine insecticide resistance monitoring activities in Benin, the susceptibility to (1) pyrethroid insecticides, (2) CTD, (3) CFP, and (4) PPF, was evaluated using WHO insecticide resistance protocols [30–34]. *Anopheles gambiae s.l.* populations (thereafter, they will be called *An. gambiae*, unless otherwise stated) with high pyrethroid resistance based on WHO bioassays [35, 36] and molecular analysis of the frequency of *kdr* gene allele, L1014F [5, 37, 38] were used in the appropriate assays for the corresponding insecticides. In Ifangni, *An. gambiae* were also exposed to Royal Guard^®^ and Interceptor^®^ G2 nets to assess the bio-efficacy of these nets on the mosquito population.

The objective of this study was to determine the susceptibility of wild pyrethroid-resistant *An. gambiae* from southern Benin to these insecticides and assess the efficacy of insecticide-treated bed nets (LLINs) that contain these new products.

## Methods

### Study sites

**Fig. 1:**
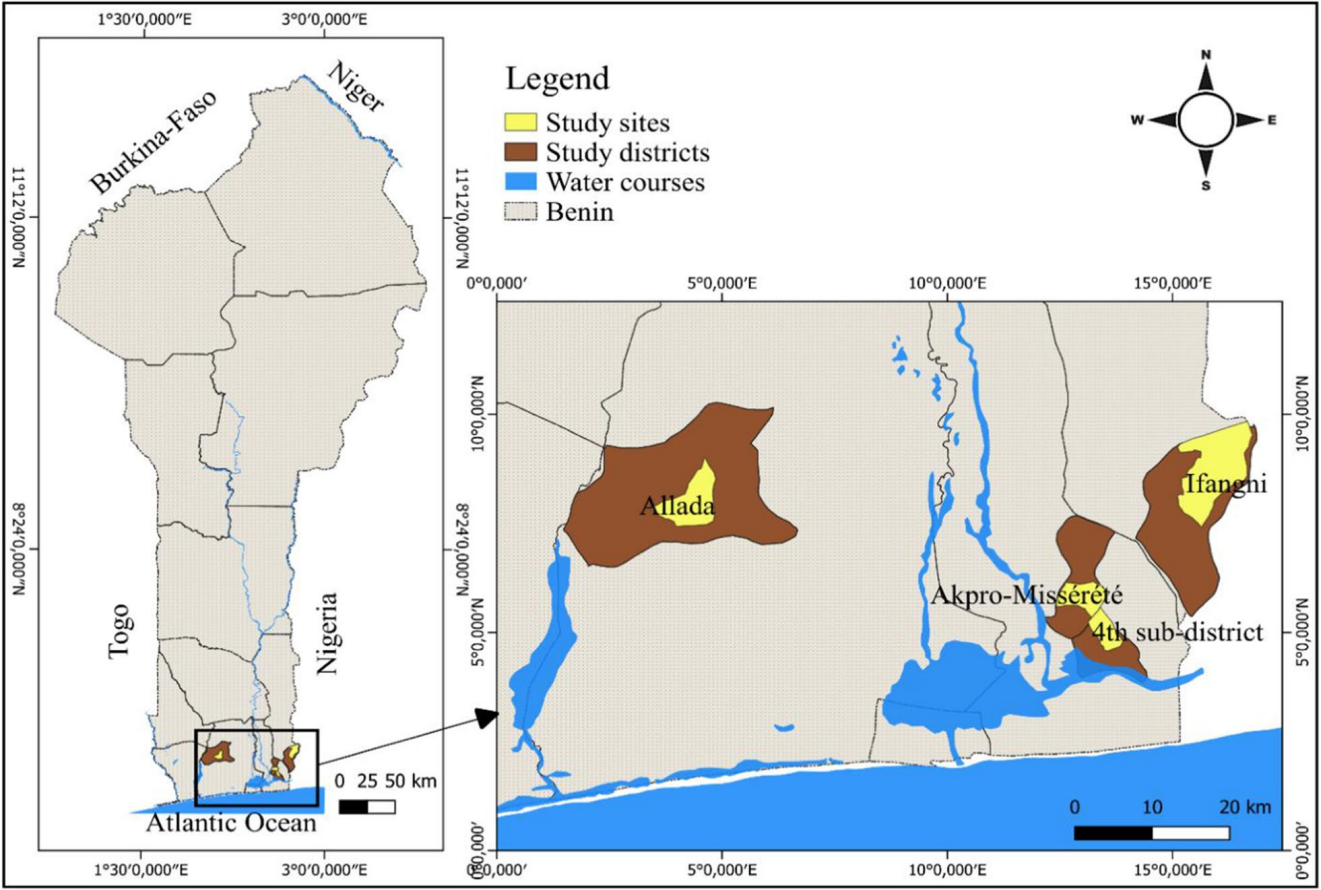
Map of study sites in Benin

This study was conducted in southern Benin from January to June 2022 in four communes: Ifangni, Akpro-Missérété, Porto-Novo, and Allada (Fig. 1). The 4 selected sites are characterized by their proximity to lagoons. The larval collection was conducted in Ita-Soumba, a village in the commune of Ifangni; Akpro-Missérété, a central arrondissement of the commune of Akpro-Missérété; Gbodjè, a neighbourhood in the fourth arrondissement of the commune of Porto-Novo; and Allada the central arrondissement of the commune of Allada. These communes were chosen based on the reported biting rate of the malaria vectors in the areas and their widespread resistance to pyrethroids in southern Benin [7, 39]. These sites have a sub-equatorial climate with two rainy seasons and two dry seasons. The communes of Ifangni and Allada are located in the Plateau and Atlantic departments, respectively, and Akpro-Missérété and Porto-Novo in the Ouémé department. Ifangni is a rural commune where agriculture, handicrafts, and commerce are the main activities. The total annual rainfall of the region is 1217.1 mm. It is crossed by the Igidi River to the east; thus, the valley of the commune is occupied by swampy forests that offer the population the opportunity to practice off-season crops, including market gardening [40]. Annual rainfall in the commune of Akpro-Missérété is between 1100 and 1300 mm, while that of Porto-Novo is about 1200 mm with very high humidity (75%). Akpro-Missérété has a few rivers, marshes, swamps, and shallows that offer the inhabitants the possibility of practicing activities such as market gardening and fish farming. In Porto-Novo, trade, fishing, and livestock are the main activities practiced [41]. In Allada, the town located in the centre of the Atlantic Department, annual rainfall averages 800 to 1000 mm. The hydrographic network is composed of the Couffo River and Lake Ahémé. The main activities are agriculture, livestock, and fishing. The LLINs distributed during mass campaigns and as part of routine campaigns (to pregnant women and schoolchildren) constitute the main means of prevention against mosquito bites in these different localities.

### Mosquito larvae collection and rearing

Larvae of *An. gambiae* were collected in the four districts of southern Benin (Allada, Ifangni, Akpro-Missérété, Porto-Novo) using standard dipping techniques [42]. The larvae were transported to the insectary of the Centre for Research in Entomology of Cotonou (CREC) for rearing. The pupae obtained were grouped in different cages and they were allowed to emerge into adult mosquitoes. After morphological identification using the Coetzee determination key [43], only specimens of *An. gambiae* were used for testing.

### Sensitivity testing of adult mosquitoes to pyrethroids, chlorfenapyr, clothianidin, and pyriproxyfen.

#### The WHO tube test for pyrethroids

Specimens of females *An. gambiae* aged 2–5 days were used for WHO tube susceptibility testing. The tests were conducted by exposing for 60 min, batches of 20–25 female mosquitoes to deltamethrin 0.05%, permethrin 0.75%, and alpha-cypermethrin 0.05% to assess the susceptibility of mosquitoes collected to these insecticides. For all tests performed, the number of mosquitoes that were knocked down by the insecticide was recorded every 15 min. Batches of 20–25 mosquitoes exposed to non-impregnated papers served as controls. Post-exposure, the mosquitoes were transferred to the observation tubes where they were fed with a 10% sugar solution for 24 h. Mortality rates were determined 24 h post-testing [4].

#### WHO tube test adapted for impregnation of filter papers with clothianidin (SumiShield^®^)

To assess the ability of a commercial formulation of an insecticide to kill a local population of field-collected *An. gambiae*, the WHO susceptibility tests with the insecticide, SumiShield^®^ 50WG, with minor modifications to the standard guidelines [33] were used. Whatman^®^ filter papers measuring 12 cm by 15 cm were treated with candidate diagnostic doses of SumiShield^®^ 50WG (containing 50% CTD) diluted in distilled water. A stock solution was prepared by diluting 264 mg SumiShield^®^ 50WG in 20 ml distilled water. Two millilitres of the mixed solution were pipetted evenly onto each filter paper and stored at 4 °C until use. Filter paper treated with 2 ml of distilled water was used as a negative control. The exposure time for CTD was set at 60 min. After exposure, mosquitoes were transferred to observation tubes lined with untreated paper (25 °C and 80% humidity) with free access to a 10% sugar solution and changed daily. The knockdown was recorded at 30 min and 60 min. Mortality was recorded at 1, 2, 3, 4, 5, 6, and 7 days after exposure [44]. This method was used before the newly finalized WHO protocols for testing clothianidin.

#### The WHO bottle bioassay with chlorfenapyr

To prepare the CFP solution for coating bottles, the stock solution was diluted with acetone. Glass Wheaton bottles (250 ml) and their caps were coated with 1 ml of 100 μg/ml of CFP. In parallel, a control bottle was coated with 1 ml of acetone, following which all bottles were covered with a sheet and left to dry in the dark for 24 h. Mosquitoes were exposed to CFP for 60 min. Following the exposure to the insecticide, mosquitoes were transferred to a paper cup covered with untreated netting, provided with lightly moistened cotton wool containing 10% sugar solution (changed daily), and monitored at 24 h, 48 h, and 72 h. The knockdown rate was recorded at 60 min and mortality at 24 h, 48 h, and 72 h after exposure. One hour after exposure to insecticide, dead mosquitoes were preserved on RNA later and stored in a − 80 freezer. Dead mosquitoes after 24 h, 48 h, and 72 h were collected and preserved in 1.5 Eppendorf tubes containing cotton and silica gel [31]. The same test was performed with a susceptible strain of *An. gambiae *sensu stricto (*s.s*.) (Kisumu) raised in an insectarium. The susceptible strain was compared with the wild mosquitoes. The testing period and 3-day post-exposure temperature (24–26 °C) were controlled using an air-conditioner. The relative humidity of the room was 78% ± 10%. This method was used before the newly finalized WHO protocols for testing chlorfenapyr.

#### Sterilizing properties of pyriproxyfen using WHO bottle bioassays

Quantification of oviposition inhibition of female *An. gambiae* requires 200 mosquitoes collected in the field and 200 mosquitoes from a susceptible laboratory colony [32]. To perform the test, 16 Wheaton^®^ 250 ml bottles with caps among which 8 bottles per mosquito strain were to be used. For each strain of mosquito, 4 bottles were covered with 1 ml of acetone (for control bottles) and 4 others with 1 ml of PPF solution of a concentration of 100 µg after dilution of the stock solution with acetone [32]. Each vial and its cap were labeled (name, insecticide concentration, date), wrapped in aluminum foil, and left open to dry for 2 h in an air condition room with a temperature between 20 °C and 23 °C. Female mosquitoes were left in a cage for one week with virile males to promote good insemination. Female mosquitoes were blood-fed once in the evening of their seventh day and a second time 48 h after the first blood meal. No later than 12 h after the second blood meal, mosquitoes were introduced in batches of 25 into the control and test vials for a one-hour exposure. They were then transferred back in batches of 25 into labeled 440-mL paper cups, fed with 10% sugar solution, and held for 72 h at 27 °C ± 2 °C and 75% ± 10% humidity where the number of live and dead mosquitoes was recorded every 24 h. After the holding period, live mosquitoes (controls and tests of both strains) were (1) individually transferred into 100 ml paper cups (oviposition chambers) covered with a piece of mosquito netting (2) provided 10% glucose solution, and (3) placed under oviposition observation for 4 days where mortality and positive (with eggs) and negative (without eggs) oviposition chambers were recorded for calculation of oviposition rate and oviposition inhibition rate. This method was used before the newly finalized WHO protocols for testing pyriproxyfen.

### Bio-efficacy Evaluation of Dual AI ITNs: Royal Guard® and Interceptor® G2

WHO cone bioassay of unwashed Royal Guard^®^ nets to determine the knockdown and mortality rate of wild populations of *An. gambiae* from Allada, Akpro-Missérété, Ifangni, and Porto-Novo.

### New-generation Interceptor G2 and Royal Guard nets were randomly selected from batches of LLINs supplied by the manufacturer for evaluation of their effectiveness at T0

Four populations of wild populations of *An. gambiae* from southern Benin (Ifangni, Akpro-Missérété, Porto-Novo, and Allada) and a susceptible laboratory strain of *An. gambiae* s.s. (Kisumu) were used to evaluate the bio-efficacy of PPF-LLIN (Royal Guard®) according to the I2I-SOP-002 protocol [45] which calls for the use of 40 mosquitoes per net (for 4 swatches of the same net). In the present study, 5 swatches of 30 cm × 30 cm of each side of unwashed Royal Guard ® LLINs were tested. A total of 50 mosquitoes of each strain were used. Two standard cones were attached with a plastic plate to each of the net swatches. Five unfed *An. gambiae* females, 2–5 days old, were introduced into each cone for 3 min. After exposure, the mosquitoes are gently removed, transferred to neutral cups, fed with sugar solution, and placed under observation for 72 h. The knockdown effect was read after 60 min of observation and mortality every 24 h until 72 h. Bioassays were performed at 25 ± 2 °C and 70 ± 10% humidity.

#### WHO cone bioassay with unwashed Royal Guard^®^ nets to determine the fertility of mosquitoes from Ifangni

**Fig. 2:**
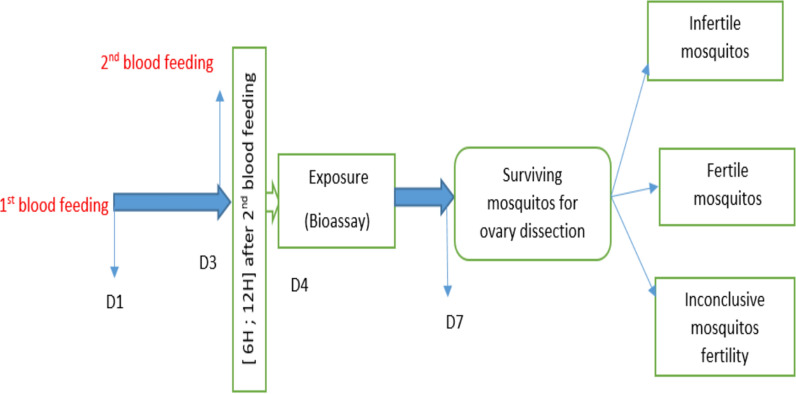
Conceptual diagram of the fertility study

The efficacy of three types of nets, unwashed PPF-LLIN (Royal Guard^®^), unwashed alpha-cypermethrin LLIN (Interceptor^®^), and negative control (unwashed untreated net) was evaluated using the WHO cone bioassay. The test was performed on adult female *Anopheles gambiae* s.l. from the Ifangni district only because of insufficient availability of mosquitoes from the larval sites from Akpro-Missérété, Allada, and Porto-Novo. Six to 12 h after their second blood meal, batches of 5 well-fed mosquitoes were exposed for 3 min to samples with two replicates of each type of net. A total of 80 mosquitoes were used per net. After exposure, mosquitoes were transferred and fed (sugar solution) in veiled cups and labeled with the net sample ID and position (roof or side). Bioassays were performed at 25 ± 2 °C and 70 ± 10% humidity. Mortality was recorded at 24, 48, and 72 h after exposure. Mosquitoes surviving 72 h after each type of net were dissected and data on the egg development status in the ovaries of each mosquito according to Christopher’s stages were reported. Fertile, infertile, and inconclusive mosquitoes were recorded on a data recording sheet and photographs of the ovaries were taken with a digital camera looking through the ocular lens (see the conceptual diagram of the study, Fig. 2).

#### Tunnel test with unwashed Interceptor^®^ G2 nets

Tunnel tests [46] were conducted only in Allada and Akpro–Missérété due to the availability of mosquitoes. Non-blood-fed 5 to 8 days old female *Anopheles* mosquitoes were released into a 60 cm square tunnel (25 cm × 25 cm square section). At each end of the tunnel, a 25 cm^2^ cage covered with a polyester net is installed. The unwashed CFP-LLIN sample was placed one-third of the way along the glass tunnel. An area of 400 cm^2^ (20 cm × 20 cm) of the net was available to the mosquitoes. Nine equidistant 1 cm diameter holes were cut into the CFP-LLIN. In the shortest part of the tunnel, an immobilized guinea pig was made available to the mosquitoes. One hundred (100) female mosquitoes were introduced into the cage at the end of the longest section of the tunnel. A separate tunnel with an untreated net was used as a negative control. After 12 to 15 h of exposure, the mosquitoes were removed from each section of the tunnel. Mortality and blood sampling rates were recorded. Inhibition of blood-feeding was assessed by comparing the proportion of blood-fed females (alive or dead) in treated and control tunnels. The overall mortality was measured by pooling the mortality rates of mosquitoes from both tunnel sections. During testing, tunnels, and cages are maintained at 27 ± 2 °C and 75% ± 10% relative humidity, at night in total darkness.

### Molecular analysis

#### Identification of Anopheles gambiae related species and frequency of kdr mutations

Since the mosquitoes showed resistance to pyrethroids, a subsample of dead and live mosquitoes obtained after exposure to deltamethrin 1 × , alphacypermethrin 1 × , and permethrin 1 × , were analyzed by PCR to determine the molecular species [47], the presence of the *kdr* L1014F mutation [48], and the presence of the G119S *ace*-1 mutation.

### Indicators and data analysis

#### Knockdown resistance (kdr) analysis

The mortality rates recorded after 24 h after exposure of mosquito populations to the diagnostic dose of the different insecticides were interpreted according to WHO criteria [30]:

- A mortality rate of ≥ 98% indicates that the tested mosquito population is susceptible.
- A mortality rate between 90 – 97% indicates the potential onset of insecticide resistance and requires further investigation.
- A mortality rate ≤ 90% indicates insecticide resistance in the tested population.

The allelic frequencies of the *kdr* L1014F mutation were determined by the following formula:

$${\varvec{F}}=\frac{2{\varvec{R}}{\varvec{R}}+{\varvec{R}}{\varvec{S}}}{2({\varvec{R}}{\varvec{R}}+{\varvec{R}}{\varvec{S}}+{\varvec{S}}{\varvec{S}})}$$

where RR is the number of homozygous mosquitoes, RS is the number of heterozygous resistant mosquitoes and SS is the number of homozygous mosquitoes.

The binomial exact test was used to test the frequencies of the *kdr* L1014F mutation and calculate confidence intervals for mortality rates. The Mann–Whitney U test was used to compare metabolic enzyme activity between the laboratory-susceptible strain (Kisumu) and those collected in the different areas. All statistical analyses were performed using the R.

#### Bio-efficacy of nets

Data on the bio-efficacy of nets tested with Kisumu and wild strains were analyzed according to WHO criteria with the variables knockdown (KD) after 60 min and mortality rate after 24 h.

$$ {\text{Optimal efficacy}}:{\text{ KD6}}0 \, \ge { 95}\% {\text{ or functional mortality }} \ge { 8}0\% . $$

$$ {\text{Minimal efficacy}}:{\text{ KD6}}0 \, \ge { 75}\% {\text{ or functional mortality }} \ge { 5}0\% . $$

*Susceptibility of* Anopheles gambiae to 100 µg *PPF.*

The WHO protocol was used to determine the susceptibility of *An. gambiae* to 100 µg PPF after exposure [32].

For tests to be considered valid the following conditions had to be met.

- Mortality of the control mosquitoes of the laboratory strain or the wild strain is > 20% at 72 h after exposure.
- The oviposition rate of the control mosquitoes of the laboratory strain or wild strain is ≥ 30% at the end of day 7 after 1 h of pyriproxyfen exposure.
- Oviposition inhibition of the susceptible laboratory strain at the end of day 7 after 1 h of exposure to pyriproxyfen is ≥ 98%.

The interpretation of the test results was as follows:

- Confirmed Resistance: a wild vector population is considered resistant to PPF if oviposition inhibition is < 90% at the end of day 7 after 1 h of exposure to the discriminating concentration of the insect growth regulator and if oviposition inhibition in the susceptible mosquito strain (tested in parallel) is ≥ 98%.
- Possible Resistance: if oviposition inhibition is ≥ 90% but < 98% at the end of day 7 after 1 h of exposure to the discriminant concentration and oviposition inhibition in the susceptible mosquito strain (tested in parallel) is > 98%, resistance in the wild strain is possible but not confirmed. The test results should be confirmed by repeating the test with a new sample from the same wild population.
- Susceptibility: a wild vector population is considered susceptible to PPF if oviposition inhibition after 1 h of exposure to the discriminating concentration is ≥ 98% at the end of day 7 and oviposition inhibition in the susceptible laboratory mosquito strain (tested in parallel) is ≥ 98%.

#### Oviposition rate

The indicators below were used to describe the oviposition rate.

- The oviposition rate (egg-laying rate) is the proportion of females that lay eggs out of the total number of females chambered after the 72-h waiting period:
- $$Oviposition\,(\% )\, = \frac{Number\,of\,females\,that\,laid}{{Total\,number\,of\,females\,initially\,chambered}} \times 100$$
- The reduction in the oviposition rate (egg-laying inhibition rate) is calculated by dividing the percentage reduction in the oviposition rate in treated females by the percentage reduction in the oviposition rate in control females:
- $$Oviposition{\mkern 1mu} \,in\,hibition\,{\mkern 1mu} (\% ){\mkern 1mu}  = {\mkern 1mu} \left[ {1 - \left( {\frac{{Oviposition\,{\mkern 1mu} \%\, {\mkern 1mu} in\,{\mkern 1mu} treatment}}{{Oviposition{\mkern 1mu} \% \,in{\mkern 1mu} control}}} \right)} \right] \times 100$$

#### Classification of eggs according to Christopher’s stages

Mosquito eggs stages were classified under a microscope and classified as follows:

- Fertile: eggs of *An. gambiae* females have fully developed to Christopher's stage V (normal, boat/sausage-shaped eggs with floats)
- Infertile: the eggs of *An. gambiae* females are not fully developed and remain in Christopher's stages I to IV (less elongated, round eggs without floats)
- Inconclusive: if both stages IV and V are observed, they are inconclusive [49].

#### Efficacy criteria in WHO cone bioassay or tunnel test

The WHO cone bioassay with Royal Guard LLIN ^®^ or tunnel test with Interceptor ^®^ G2 ITNs was used to determine the efficacy of ITNs used in assays. Using Phase I study protocols [50] for the evaluation of LLIN s, nets were considered effective against the *An. gambiae* populations, if mosquitoes from bioassays had ≥ 80% mortality or ≥ 95% knockdown or mosquitoes in tunnel test, had ≥ 80% mortality or ≥ 90% blood-feeding inhibition.

## Results

### Identification of *Anopheles gambiae* from the four areas of southern Benin

**Fig. 3:**
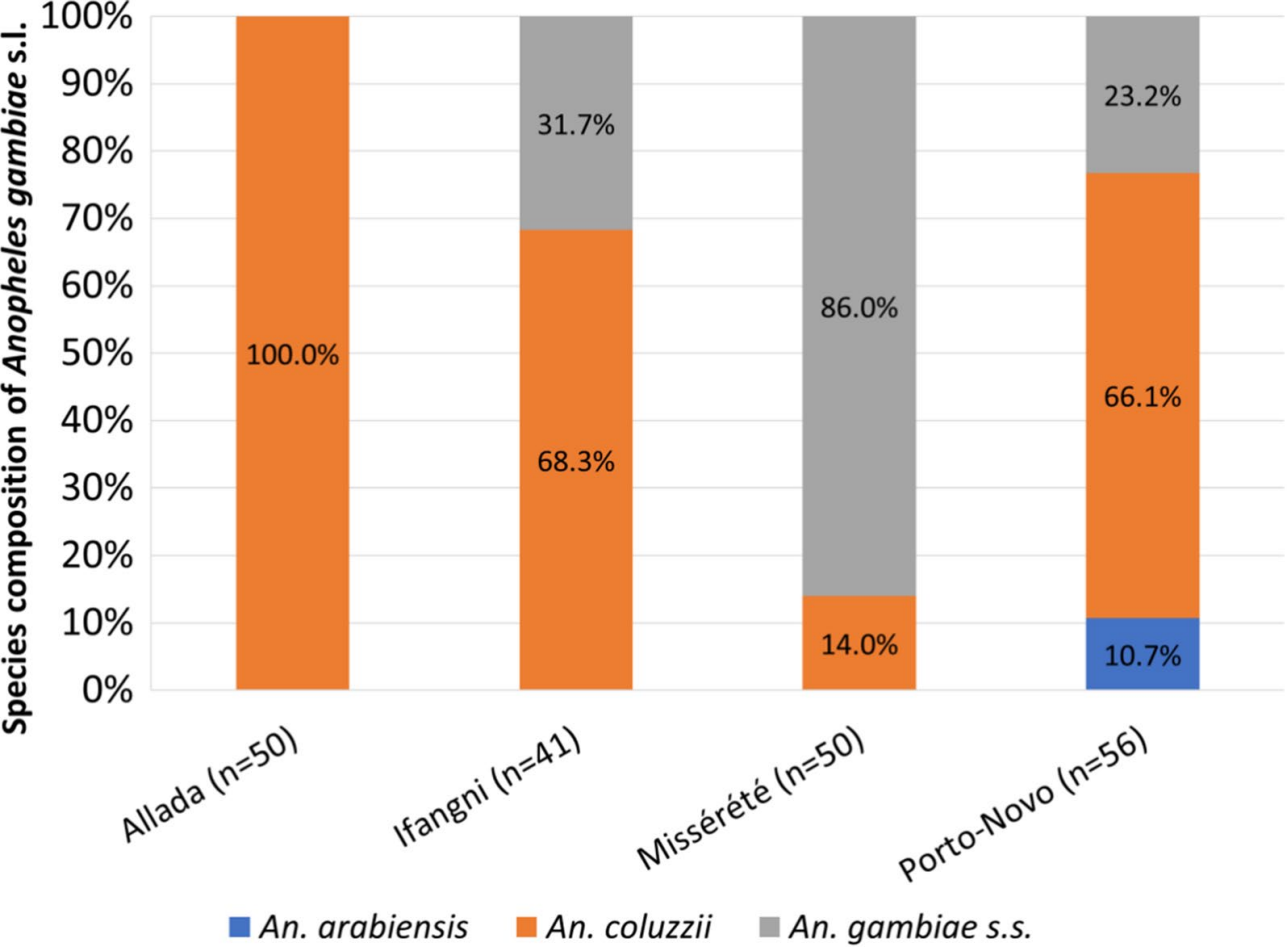
Percentage (%) species composition of *Anopheles gambiae* in Allada Ifangni, Akpro-Missérété, and Porto-Novo

Molecular species identification of the *An. gambiae* complex from the 4 sites predominantly identified *Anopheles coluzzii* in Allada (100%), Ifangni (68.3%), and in Porto-Novo (66.1%), while *An. gambiae s.s.* was predominately identified in Akpro-Missérété (86%). A relatively small proportion of *Anopheles arabiensis* (10.7%) was identified in Porto-Novo only (Fig. 3).

### Susceptibility testing of adult mosquitoes to pyrethroids, clothianidin, chlorfenapyr, and pyriproxyfen from the four areas of southern Benin

#### Susceptibility of *An. gambiae* to permethrin, alpha-cypermethrin, and deltamethrin using the WHO tube test

**Fig. 4:**
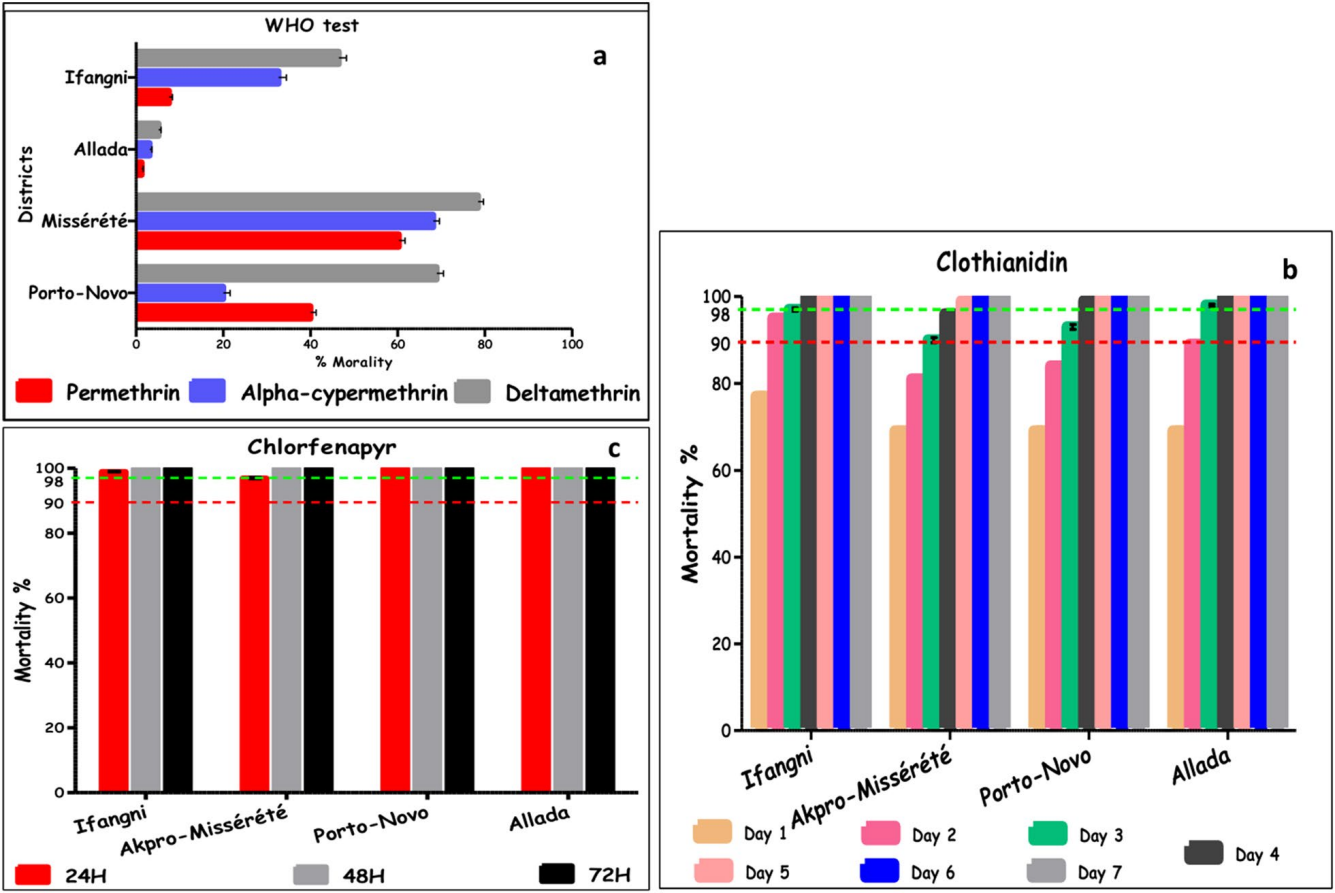
Mortality rate of *Anopheles gambiae* from the lagoon areas in Ifangni, Allada, Akpro-Missérété, and Porto-Novo after exposure to **a** permethrin, deltamethrin, and alpha-cypermethrin using the WHO tube test; **b** clothianidin using the WHO tube test with impregnated papers with SumiShield^®^; and **c** chlorfenapyr using the WHO bottle bioassay

Monitoring vector resistance to insecticides was carried out in lagoonal areas in 4 districts in southern Benin (Porto-Novo, Akpro-Missérété, Allada, and Ifangni) with three pyrethroids (deltamethrin, permethrin, and alpha-cypermethrin) according to the WHO tube test (Fig. 4a). The purpose of this monitoring was to assess the resistance status of the four populations of *An. gambiae* to pyrethroids. Mortality rates were below 80% for all insecticides and locations indicating a high resistance to these insecticides.

The frequency of the L1014F allele of the *kdr* gene was high at all sites (> 0.70), while the frequency of the G119S allele of the *ace*-*1R* gene was very low (< 0.03). Statistical analyses revealed that there was no significant difference between the frequencies of the *kdr* and *ace-1* resistance genes in the different study sites.

#### Susceptibility of *An. gambiae* to clothianidin (CTD) using the WHO tube test and impregnated filter paper with SumiShield^®^

Using impregnated filter papers with SumiShield^®^, the mortality rates observed 24 h after exposure to CTD were below 80% in all districts: 77% in Ifangni and 69% in Akpro-Missérété, Porto-Novo, and Allada. The mortality rate increased progressively to reach 95% in Ifangni after 48 h while in the other districts, it remained below 90%. The susceptibility threshold was reached after 72 h in Allada (98%), and after 96 h in Porto-Novo (99%) and Ifangni (100%). The total susceptibility (100%) was observed on the fifth day in Ifangni, Allada, and Porto-Novo, and on the sixth day in Akpro-Missérété (Fig. 4b).

#### Susceptibility of *An. gambiae* to chlorfenapyr (CFP) using the WHO bottle bioassay

The four populations of *An. gambiae* (Ifangni, Akpro-Missérété, Porto-Novo, Allada) were all susceptible to CFP, 24 h after the test, the mortality rates observed were 100% in Porto-Novo and Allada, 99% in Ifangni, and 97% in Akpro-Missérété. After 48 h, the mortality rate increased to 100% in all the localities tested. (Fig. 4c).

#### Mortality and sterilizing effects of 100 µg pyriproxyfen (PPF) on An. gambiae from Ifangni using the WHO bottle bioassay

The mortality rate of the different strains of *An. gambiae* (wild population and Kisumu susceptible strain) exposed to PPF and acetone was monitored for 72 h. This rate did not exceed 20% for any of the strains. For the wild population tested, the mortality rate was 4.1% [95% confidence interval: 1.1–10.2] with PPF and 11.34% [95% confidence interval: 5.8–19.4] with acetone. For the susceptible laboratory Kisumu strain, the mortality rate was 10.0% [95% confidence interval: 4.9–17.6] with PPF and 2.1% [95% confidence interval: 0.3–7.5] with acetone (Table 1).

**Table 1:** Mortality rate of a susceptible laboratory colony of *Anopheles gambiae* s.l. (Kisumu strain) and wild *Anopheles gambiae* s.l. from Ifangni at 24-, 48-, and 72-h post-exposure to 100 μg pyriproxyfen (PPF) and acetone (control) using the WHO bottle bioassay

Strain	Insecticide	Number tested	Percentage (%) mortality at different time points [95% confidence interval]		
			24 h	48 h	72 h
Ifangni	PPF	97	1.0 [0–5.6]	2.1 [0.3–7.3]	4.1 [1.1–10.2]
	Acetone	97	3.1 [0.6–8.8]	6.2 [2.3–13]	11.3 [5.8–19.4]
Kisumu	PPF	100	8.0 [3.5–15.2]	9.0 [4.2–16.4]	10.0 [4.9–17.6]
	Acetone	94	0	0	2.1 [0.3–7.5]

**PPF**: pyriproxyfen; %: percentage

Table 2 shows the oviposition rate and oviposition inhibition rate of the wild strain Ifangni and the susceptible strain of *An. gambiae* after exposure to PPF and acetone. The oviposition rate of the wild strain Ifangni after 4 days was 1.1% [95% confidence interval: 0.03–5.8] after exposure to PPF and 79.1% [95% confidence interval: 69.0–87.1] after exposure to acetone. The oviposition rate of the susceptible strain (Kisumu) was 1.1% [95% confidence interval: 0.03–6.04] after PPF exposure and 98.9% [95% confidence interval: 94.1–100] after acetone exposure. The oviposition inhibition rate calculated from the oviposition rates showed that the wild population of *An. gambiae* from Ifangni was 98.7% [95% confidence interval: 93.1–100] and the susceptible Kisumu strain was 99.0% [95% confidence interval: 94.4–100]; both reached the threshold of susceptibility to PPF.

**Table 2:** Oviposition and oviposition inhibition rates of a susceptible laboratory colony of *Anopheles gambiae* s.l. (Kisumu strain) and wild *Anopheles gambiae* s.l. from Ifangni after exposure to 100 μg pyriproxyfen (PPF) and acetone (control) using the WHO bottle bioassay

Strain	Test	Chambers tested at 96 h	Percentage (%) of eggs laid at different time intervals [95% confidence interval {CI}]				Oviposition inhibition (%) [95% CI]
			96 h	120 h	144 h	168 h	
Ifangni	PPF	93	0	0	1.1 [0.03–5.8]	1.1 [0.03–5.8]	98.7 [93.1–100]
	Acetone	86	0	14.0 [7.4–23.1]	61.6 [50.5–71.9]	79.1 [69.0–87.1]	
Kisumu	PPF	90	0	0	1.1 [0.03–6.0]	1.1 [0.03–6.0]	99.0 [94.4–100]
	Acetone	92	66.3 [55.7–75.8]	82.6 [73.3–89.7]	87.0 [78.3–93.1]	98.9 [94.1–100]	

Hrs: hours; PPF: pyriproxyfen

### Bio-efficacy evaluation of dual AI ITNs: Royal Guard^®^ and Interceptor^®^ G2

#### WHO cone bioassay with unwashed Royal Guard^®^ nets (PPF-LLIN) using Kisumu strain mosquitoes and wild mosquitoes from Allada, Akpro-Missérété, Ifangni, and Porto-Novo

**Fig. 5:**
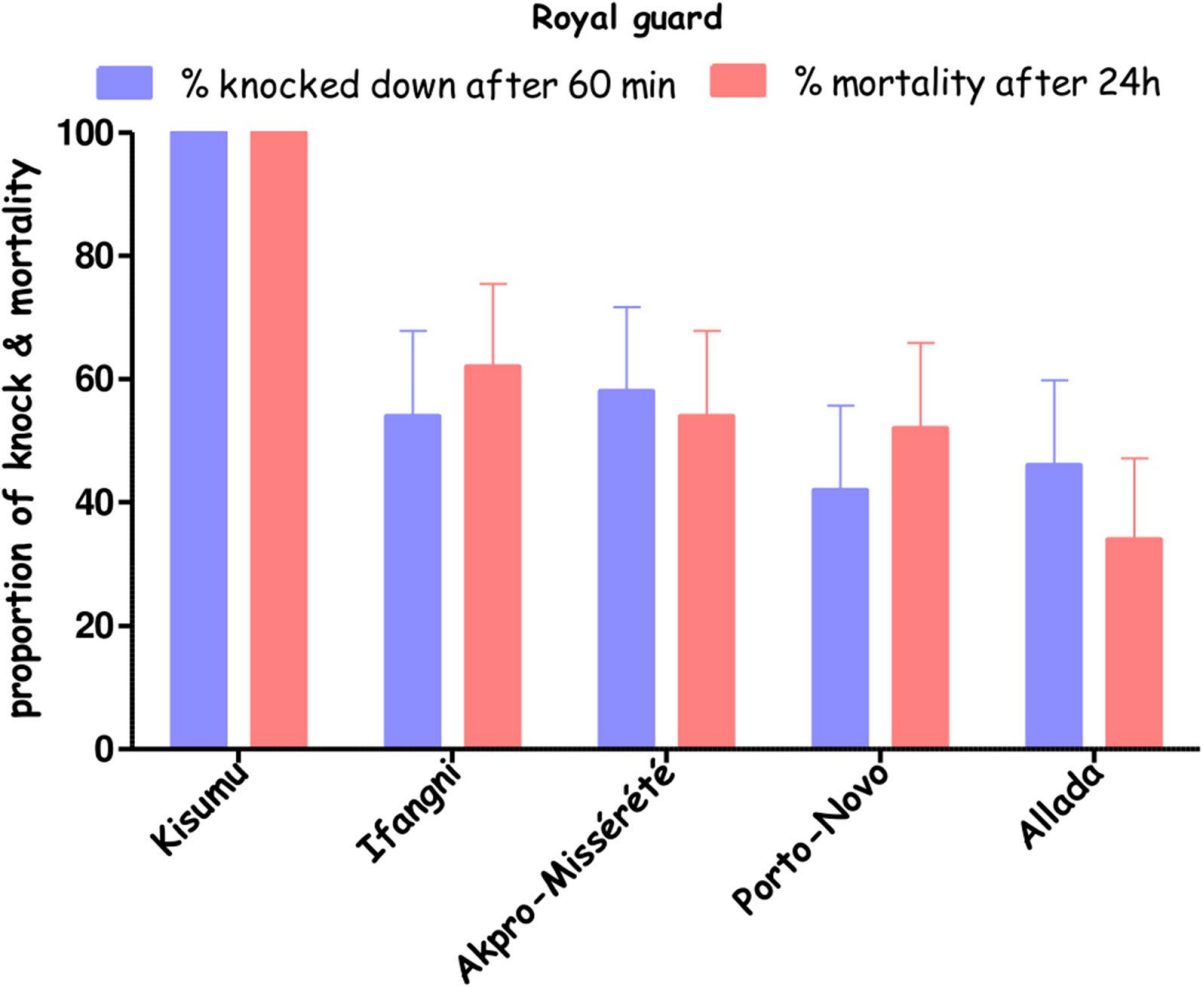
Knockdown and mortality rate of *Anopheles gambiae* from lagoon areas in Ifangni, Allada, Akpro-Missérété, and Porto-Novo after exposure to a Royal Guard® Net using the WHO cone bioassay on nets

The knockdown percentage and mortality rate observed 24 h after exposure of the Kisumu strain to the unwashed PPF-LLIN was 100%. For the wild populations of *An. gambiae*, the knockdown rates ranged from ~ 41% in Porto-Novo to ~ 59% in Akpro-Missérété, and the mortality rates ranged from ~ 34% in Allada to ~ 62% in Ifangni (Fig. 5).

#### Impact of unwashed PPF-LLIN (Royal Guard^®^) on the fertility of *An. gambiae* from Ifangni using the WHO cone bioassay

Table 3 presents the results of the fertility of females of *An. gambiae* mosquitoes from Ifangni were exposed to 3 types of nets: unwashed Royal Guard, unwashed Interceptor^®^ (positive control), and an unwashed untreated net (negative control). All mosquitoes exposed to the Royal Guard nets were infertile with no egg follicle reaching Christopher’s stage IV or V), while 74.2% [95% confidence interval: 55.4–88.1] of *An. gambiae* exposed to the Interceptor^®^ net and 73.8% [95% confidence interval: 58.0–86.1] exposed to the untreated net had fully developed egg follicles (i.e., Christopher’s stage V). Finally, 3.2 percent [95% confidence interval: 0.1–16.7] of mosquitoes exposed to the Interceptor^®^ net and 21.4% [95% confidence interval: 10.3–36.8] of mosquitoes exposed to the untreated net had inconclusive results.

**Table 3:** Impact of new unwashed Royal Guard^®^, Interceptor^®^, and untreated nets on the fertility of *Anopheles gambiae* s.l. from Ifangni using the WHO cone bioassay

Mosquito nets	Mosquito number tested	Egg development Christopher’s stage						Fertility status (%) [95% confidence interval]		
		I	IIa	IIb	III	IV	V	Fertile	Infertile	Inconclusive
Untreated net	42	0	0	1	1	9	31	73.8 [58.0–86.1]	4.8 [0.6–16.2]	21.4 [10.3–36.8]
Royal® Guard	14	5	3	5	1	0	0	0	100 [76.8–100]	0
Interceptor®	31	0	0	3	4	1	23	74.2 [55.4–88.1]	22.6 [9.6–41.1]	3.2 [0.1–16.7]
Total	87	5	3	9	6	10	54	62.1 [51.0–72.3]	26.4 [17.6–37.0]	11.5 [5.7–20.1]

*%* percentage

#### Efficacy of unwashed CFP-LLIN (Interceptor^®^ [IG2]) on wild populations of *An. gambiae* from Allada and Akpro-Missérété using the WHO tunnel test

Table 4 presents the results of the tunnel test after exposure of wild populations of *An. gambiae* in Allada and Akpro-Missérété to an IG2 net. Mortality after 15 h of exposure was 3% [95% confidence interval: 0.6–8.5] and 75% [95% confidence interval: 65.3–83.1], respectively for the control net and IG2 net in Allada; 1% [95% confidence interval: 0.03–5.4) and 65% [95% confidence interval: 54.8–74.3], respectively for the control net and IG2 net in Akpro-Missérété. The penetration rate was 43.0% [95% confidence interval: 33.1–53.3] and 14.0% [95% confidence interval: 7.9–22.4] for the control net and IG2 net, respectively in Allada; 27.0% [95% confidence interval: 18.6–36.8] and 18.0% [95% confidence interval: 11.0–26.9] for the control net and IG2 net, respectively in Akpro-Missérété. The blood-feeding rate was 42% [95% confidence interval: 32.2–52.3] for the control net and 4% [95% confidence interval: 1.1–9.9] for the IG2 net in Allada; and 25% [95% confidence interval: 16.9–34.7] and 2% [95% confidence interval: 0.2–7.0] in Akpro-Missérété. The blood feeding inhibition rate of 90.1% [95% confidence interval: 77.4–97.3] in Allada and 92.0% [95% confidence interval: 73.9–99.0] in Akpro-Missérété met the threshold for net efficacy in the Phase 1 studies.

**Table 4:** Mortality, penetration, and blood–feeding inhibition rates of wild populations of *Anopheles gambiae* s.l. after exposure to a new unwashed Interceptor G2^®^ (IG2) net using the WHO tunnel test

Site	Mosquito net	Number tested	Mortality (%) [95% CI]	Penetration (%) [95% CI]	Blood-fed (%) [95% CI]	Blood-feeding inhibition (%) [95% CI]
Allada	Untreated net (Control)	100	3.0 [0.6–8.5]	43.0 [33.1–53.3]	42.0 [32.2–52.3]	90.1 [77.4–97.3]
	IG2	100	75.0 [65.3–83.1]	14.0 [7.9–22.4]	4.0 [1.1–9.9]	
Akpro–Missérété	Untreated net (Control)	100	1.0 [0.03–5.4]	27.0 [18.6–36.8]	25.0 [16.9–34.7]	92.0 [73.9–99.0]
	IG2	100	65.0 [54.8–74.3]	18.0 [11.0–26.9]	2.0 [0.2–7.0]	

*%* percentage, *CI* 95% confidence intervals

## Discussion

The potential efficacy of clothianidin, chlorfenapyr, and pyriproxyfen was assessed against wild pyrethroid-resistant *An. gambiae* populations from four southern areas of Benin (Allada, Akpro-Missérété, Ifangni, and Porto-Novo). This study demonstrates that pyrethroid-resistant *An. gambiae* from select communities of southern Benin were fully susceptible to clothianidin and chlorfenapyr (≥ 98% mortality), and pyriproxyfen was effective in inhibiting oviposition of these pyrethroid-resistant *An. gambiae* (≥ 98% oviposition inhibition) using the WHO bottle bioassay. This study also demonstrates that new unwashed Interceptor^®^ G2 nets were able to inhibit blood-feeding by ≥ 90% and new unwashed Royal Guard^®^ nets were able to completely sterilize the wild pyrethroid-resistant *An. gambiae*. However, it is important to note that WHO bottle bioassays with pyriproxyfen, WHO cone bioassays with Royal Guard^®^ nets, and WHO tunnel test with Interceptor^®^ G2 nets were not done with mosquitoes from all 4 sites due to the limited number of mosquitoes to do the different assays. Nonetheless, these results provide insights into the potential impact of these insecticides and nets in select sites in southern Benin. In addition, the limited geographical coverage (only 4 sites surveyed in Southern Benin) is a limitation for the present study.

The four populations of *An. gambiae* (Ifangni, Akpro-Missérété, Allada, Porto-Novo) were found to be fully susceptible to 2% CTD. In a study, conducted in India indoor residual spraying with CTD was found to be effective, operationally feasible, safe, and effective for up to 6 months [21]. A similar result was found in a recent study conducted in Benin showing that CTD used in a spray campaign showed the potential to provide prolonged control of malaria transmitted by pyrethroid-resistant mosquito populations for up to 8–9 months [18]. During a campaign of Indoor Residual Spraying using SumiShield 50WG (CTD 300 CS) in Alibori and Donga (northern Benin), Odjo et al. (2021) showed a decrease in entomological inoculation rate (EIR) of *An. gambiae* (unpublished). During this trial, bioassays with a laboratory strain of susceptible *An. gambiae* s.s. (Kisumu) showed that SumiShield® 50 WG remains effective with a mortality rate of over 80% on treated walls and 75% of the wild field strain, five months after the spraying date and after 24 h of observations. One year before, bioassays on treated walls carried out in the same region with Fludora ® Fusion (mixture of clothianidin and deltamethrin) have shown that this formulation remains effective with a mortality rate above 90% on the susceptible strain Kisumu, 4 months after spraying date (Odjo et al. 2020, unpublished). During this period of bio-efficiency of Fludora ^®^ Fusion, the authors observed a high reduction of some indicators like the indoor resting density, sporozoite index, and EIR and a strong exophagy of *An. gambiae* in most treated districts compared to control areas.

In this study, the results of the WHO bottle bioassay with CFP were like other studies [18, 23, 47, 51–54], which have demonstrated strong efficacy against wild pyrethroid-resistant *An. gambiae*. The results of the WHO tunnel experiment with Interceptor® G2 nets in this study also align with other studies, which have shown that Interceptor^®^ G2 has a significant impact on mortality, penetration (i.e., LLIN avoidance), and blood-feeding success. Ngufor et al*.* [18] showed, in a study conducted in Covè (southern Benin), that indoor residual spraying with CFP in households with standard LLINs provided additional levels of transmission control and personal protection thus revealing the potential of CFP to manage pyrethroid resistance in the context of an expanding LLIN/IRS strategy. Recently, there have been two studies, one in Benin [55] and one in Tanzania [29], which have demonstrated a high reduction in human malaria indicators in the Interceptor® G2 net arm compared to the standard pyrethroid-only net arm in randomized control trials after two years of use. All these studies continue to provide evidence of the potential efficacy of chlorfenapyr-based products for vector control. However, the mosquito penetration and feeding rates in the untreated control nets were below the minimum threshold of 50% required in the tunnel tests by the WHO [50]. This could be explained by the fact that guinea pigs were less attractive to wild mosquitoes. This is a limitation for the study.

The results of several studies have effectively shown the ability of pyriproxyfen to inhibit the development of *An. gambiae* larvae [14, 56–58]. The use of pyriproxyfen has been expanded to adult female *An. gambiae* and significantly impairs reproduction [15, 59]. In this study, the oviposition inhibition rate (98.6%) for the wild population of *An. gambiae* from (Ifangni) had reached the WHO-threshold definition of susceptibility to PPF. Furthermore, the sterilizing impact of female *An. gambiae* exposed to PPF was observed where all mosquitoes exposed to Royal Guard® net were infertile with no eggs reaching Christopher’s stage V while most eggs of *An. gambiae* exposed to the control nets had fully developed their eggs to Christopher’s stage V. While these study results would suggest that Royal Guard^®^ or other pyriproxyfen-based products may potentially lead to a significant reduction in malaria transmission, studies in randomized control trials in Tanzania [29] and Benin [55] have demonstrated that Royal Guard^®^ LLINs show no greater impact on epidemiological indicators than standard pyrethroid only LLINs.

The distance between Ifangni and the study sites in the Benin randomized control trial (Covè, Zagnanado, and Ouinhi communes) [55] is between ~ 75 and ~ 100 km apart. The primary vectors, *An. coluzzii,* and *An. gambiae* s.s., in these areas have high pyrethroid resistance intensity [28, 60]. Post-intervention insecticide resistance assays from the Benin randomized control trial further showed that exposure of *An gambiae*, from the study area to pyriproxyfen led to a high reduction in the fecundity rate (over 70%) over the 2 years after the net distribution, compared to unexposed control mosquitoes [55]. In this study, the oviposition inhibition rate of *An. gambiae* from Ifangni after exposure to pyriproxyfen was 98.7%, which could suggest a greater impact of pyriproxyfen LLINs in this area. It is not clear if geographical variation in fecundity impairment after laboratory exposure to pyriproxyfen would translate into variable entomological and epidemiological outcomes after the deployment of pyriproxyfen LLINs. Further investigation would be required to determine this.

It has been suggested that while pyriproxyfen does seem to reduce entomological indicators of malaria transmission, this reduction may not be large enough to translate into a reduction in human epidemiological indicators of malaria transmission [29, 55]. Given these results, it is not clear what role pyriproxyfen should play in vector control. Perhaps, pyriproxyfen should be coupled with alternative insecticide classes since wild adult populations of *An. gambiae* has established widespread resistance to pyrethroids. Rather, it may be more effective to couple pyriproxyfen with chlorfenapyr or other new LLIN insecticides to either increase efficacy or slow the onset of insecticide resistance in wild mosquito populations. However, it would be important to (1) determine if the insecticide chemistries are compatible to remain on LLINs; (2) determine if the insecticide can work together synergistically to either reduce vectorial capacity, slow insecticide resistance to either insecticide, or both; and (3) determine if the combination of these two insecticides are more effective than other dual AI combinations that include pyrethroids. Alternatively, given pyriproxyfen’s original use as a larvicide, LLINs may not be optimally suited as the AI delivery method to kill mosquitoes.

While this study adds to the knowledge base of insecticide resistance in Benin some limitations are noted. It is important to note that deviations from recent WHO guidelines and SOPs are due to this occurring before the publication updates for the clothianidin and chlorfenapyr insecticides, and bio-efficacy tests. In this study, the protocol [31] using clothianidin or chlorfenapyr with methyl ester of rapeseed oil (MERO) was not used; at the time of the study, MERO was not available to the author for use in the assay. SumiShield® was impregnated on filter paper and used as the source of clothianidin in the WHO tube test [33] with the rationale that it was a stable product formulated to remain on surfaces and would provide a direct assessment of how wild mosquitoes respond to a formulated commercial product. Bottle bioassays were coated with chlorfenapyr without MERO. It is currently not recommended to use WHO cone bioassays for dual AI LLINs. It is not clear how these test deviations may have affected the measurement of insecticide resistance. While these results may still suggest the susceptibility of these four populations to the insecticides, it will be important to repeat the tests with clothianidin and chlorfenapyr using the appropriate protocols. In this study, only new unwashed LLINs were used in the WHO cone bioassay and the WHO tunnel test. Other studies have reported washed and unwashed nets to be similar in bioassay [22]. Therefore, this study is still useful in understanding the response of *An. gambiae* to new insecticides and LLINs. In the WHO bottle bioassays assessing oviposition inhibition rates, a pyrethroid exposure group was not included in the assay for comparison. Finally, only Ifangni was assessed for oviposition and fertility inhibition, and only Allada and Akpro–Missérété were assessed using Interceptor^®^ G2 LLINs. However, while a pyrethroid group would have been useful to include for comparison the main goal of this assay was to assess the susceptibility of the mosquitoes to pyriproxyfen. Sourcing enough mosquitoes from all the sites for each assay was a challenge. This is a typical problem when trying to conduct multiple assays using wild-caught material. Nonetheless, this study still provides useful information to understand the value of these tools for malaria control in the selected study sites in southern Benin.

## Conclusion

The results of this study confirm the entomological efficacy of CFP, CTD, and PPF against wild pyrethroids*-*resistant *An. gambiae* in Benin, where mosquito populations from the four lagoon areas of southern Benin were found to be susceptible to these insecticides. Furthermore, nets treated with a combination of CFP (Interceptor® G2) or PPF (Royal Guard^®^) and ACM also have good efficacy against these populations with PPF-treated nets significantly inducing sterility in wild populations of *An. gambiae* and CFP-treated nets inducing significant blood-feeding inhibition. This study along with other studies shows that under laboratory conditions chlorfenapyr and pyriproxyfen may be effective insecticides against pyrethroid-resistant mosquitoes. Observational entomological monitoring studies which investigate entomological and epidemiological indicators after the deployment of these LLINs could provide a better understanding of the effectiveness of these products. Nonetheless, despite the availability of new effective insecticides, continued vigilance is needed in Benin through routine insecticide resistance monitoring to periodically update Benin’s national insecticide resistance database and management plan.

## Data Availability

The data used and/or analyzed in this study are available from the corresponding author upon reasonable request.

## Abbreviations

*ace-1*: Acetylcholinesterase-1
AI: Active ingredient
CDC: Centers for Disease Control and Prevention
CFP: Chlorfenapyr
CREC: Centre de Recherche Entomologique de Cotonou
CTD: Clothianidin
IG2: Interceptor G2® IRS: indoor residual spraying
LLIN: Insecticide-treated net; *kdr*: knockdown resistance
MERO: Methyl ester of rapeseed oil
NMCP: National Malaria Control Programme
PCR: Polymerase chain reaction
PMI: U.S. President’s Malaria Initiative
s.l.: Sensu lato
s.s.: Sensu stricto
PPF: Pyriproxyfen
USAID: United States Agency for International Development
WHO: World Health Organization
